# Electrical impedance tomography-guided positive end-expiratory pressure titration for perioperative oxygenation and postoperative pulmonary complications: A systematic review and meta-analysis

**DOI:** 10.1097/MD.0000000000040357

**Published:** 2024-12-27

**Authors:** Lifang Chen, Kang Yu, Jiaojiao Yang, Xue Han, Lei Liu, Tianzuo Li, Huihui Miao

**Affiliations:** a Department of Anesthesiology, Beijing Shijitan Hospital, Capital Medical University, Beijing, China; b Department of Science and Technology, Beijing Shijitan Hospital, Capital Medical University, Beijing, China.

**Keywords:** electrical impedance tomography, meta-analysis, postoperative pulmonary complications

## Abstract

**Background:**

The electrical impedance tomography (EIT)-guided individual positive end-expiratory pressure (PEEP) approach is a noninvasive, radiation-free, and straightforward strategy. However, its validity to prevent postoperative complications remains unclear. To determine whether the EIT-guided PEEP titration in surgery has a higher oxygenation index and lower postoperative complications incidence in patients, we performed a meta-analysis to assess the efficacy. The study design is a systematic review and meta-analysis.

**Methods:**

Four databases (Cochrane, PubMed, Web of Science, and Embase) were searched from 2000 to November 2022 for this study. Randomized controlled trials of patients selected for general anesthesia were included. The main indicators of the study were oxygenation and postoperative pulmonary complications. Study quality was assessed using the Cochrane Risk and Bias Tool.

**Results:**

A total of 7 articles with 425 subjects were included and were eligible for analysis. Meta-analysis showed that patients had a higher oxygenation index (PaO_2_/FiO_2_) after EIT-guided individual PEEP titration compared with other modalities of PEEP titration (6 trials, 351 subjects, standardized mean check = 1.06, 95% confidence interval = 0.59–1.53). For subgroup analysis, the results were still statistically significant both in adult/elder groups and normal/obese groups. No significant advantage was found for the incidence of postoperative pulmonary complications between individual PEEP titration under EIT and other titration strategies (5 trials, 341 subjects, standardized mean check = 0.77, 95% confidence interval = 0.34–1.71). The same results were found in the subgroup analysis.

**Conclusion:**

EIT-guided individual PEEP setting significantly improved perioperative oxygenation index compared with other modalities of PEEP ventilation strategies for patients, but no significant differences were found in the incidence of the postoperative pulmonary complications.

## 1. Introduction

Invented in the 1980s, electrical impedance tomography (EIT) is a noninvasive, radiation-free imaging technique based on the different electrical conductivities of human tissues.^[1]^ EIT has promising clinical applications in adults, including real-time monitoring of mechanical ventilation and cardiopulmonary perfusion. The effectiveness of EIT has been confirmed by studies that stem from several previous trials, including CT,^[2–6]^ positron emission tomography.^[7]^ EIT is characterized by the ability to perform continuous region-specific measurements of the lung in a dynamic situation and is used to analyze the heterogeneity of lung tissue in specific regions. An increasing number of studies have reported the feasibility of the EIT technique in the perioperative period, which is an important guide for preoperative lung function assessment, intraoperative individualized mechanical ventilation management, and postoperative pulmonary complication management.^[8]^

The correct setting of intraoperative positive end-expiratory pressure (PEEP) plays an important role in preventing the development of pulmonary atelectasis. Insufficient PEEP cannot maintain alveolar expansion, while too much PEEP may lead to lung injury. EIT can accurately measure whole-lung and regional lung ventilation distribution, and individualized PEEP settings based on EIT have become a hot spot for research. During abdominal surgery anesthesia, while receiving protected tidal volume ventilation, EIT is used to guide PEEP titration to determine the nearest PEEP value above the intersection of the lung hyperdilation and collapse curves as the optimal PEEP, which can reduce the incidence of postoperative pulmonary atelectasis.^[9]^ Reduces the probability and severity of pulmonary atelectasis, decreases driving pressures, and improves respiratory compliance and oxygenation.

In addition to abdominal surgery, EIT for patients at high risk for postoperative pulmonary complications such as obesity and structural lung disease should be used for respiratory management throughout the perioperative period. Preoperative application of EIT in patients with COPD and asthma allows dynamic assessment of the severity of expiratory flow limitation and efficacy to bronchodilators to guide the setting and dosing of perioperative PEEP.^[10,11]^ Morbidly obese patients often have significantly reduced functional residual air volume during induction of anesthesia due to decreased respiratory muscle tone and thoracoabdominal fat compression, which can lead to severe hypoxemia.^[12]^ The application of EIT for postoperative respiratory management is relatively mature, but in the future it is necessary to emphasize the timing of individualized respiratory management forward to achieve the whole process of perioperative respiratory management with the aim of reducing the incidence and severity of postoperative pulmonary complications and severity of postoperative pulmonary complications.

The advantage of our trial is that in addition to abdominal surgery, procedures at high risk for postoperative pulmonary complications such as thoracoscopy and surgery in obese patients were included. Previous studies have used EIT-guided individual PEEP titration with other modality titration strategies for postoperative testing and acute respiratory distress syndrome (ARDS). Our study has compared EIT with other PEEP titration strategies applied to perioperative mechanical ventilation to compare patients’ perioperative oxygenation indices and the incidence of postoperative pulmonary complications. The study in this paper is characterized by the inclusion of surgical procedures including abdominal surgery and orthopedic surgery, among other surgical types, which include different body positions.

## 2. Methods

We searched 4 electronic databases (Pubmed; Embase; Web of Science; Cochrane) from the date of establishment to November 2022 for studies of alternative end-expiratory orthostatic pressure titration strategies for patients undergoing general anesthesia and individual EIT-based end-expiratory orthostatic pressure settings, using MeSH headings and text words (for a detailed discussion of the search strategy, Appendix, Supplemental Digital Content, http://links.lww.com/MD/O230). We searched the references of all identified publications for additional studies, including previous Meta-analyses and reviews. This trial is registered with PROSPERO (ID: CRD42022381117).

### 2.1. Selection criteria

The inclusion criteria for this study were as follows: 1. randomized controlled trial (RCT); 2. patients were adults over 18 years old under general anesthesia; 3. EIT-guided PEEP titration was used in the experimental group, and other PEEP settings were used in the control group; 4. the final outcome metrics included the Oxygenation index. Postoperative pulmonary complications (PPCs)^[13]^ including: (I) atelectasis detected on CT scans or chest radiographs; (II) pneumonia diagnosed according to the Centers for Disease Control standards^[14]^ or aspiration pneumonia; (III) acute respiratory distress syndrome diagnosed according to the Berlin definition^[15]^; (IV) stump leakage, chest drainage for more than 5 days; (V) bronchopleural fistula; (VI) ventilation support for more than 48 hours; (VII) re-tracheal intubation; (VIII) empyema; (IX) respiratory failure; (X) pulmonary embolism; and (XI) pleural effusion.

Exclusion criteria were as follows: 1. data that could not be extracted; 2. duplicate studies; 3. reviews, conference articles and editorials; and 4. animal experiments.

### 2.2. Data extraction

All articles were searched by 1 researcher. Two additional researchers independently screened articles based on inclusion and exclusion criteria. Two researchers independently assessed their eligibility and extracted demographic and outcome data for each study into a standardized table. If disagreements were encountered, a third reviewer was consulted to make the final decision. In case of missing data, we contacted the article authors by email and completed our data. This study is reported by the Preferred Reporting Items for Systematic Reviews and Meta-Analysis statement.^[16,17]^ This study was systematically evaluated using the Cochrane risk of bias assessment tool. No institutional review board approval was required for this meta-analysis because the study included data that had been published previously.

### 2.3. Statistical analysis

The analysis used odds ratio and 95% confidence intervals (CIs) to report dichotomous outcomes (postoperative pulmonary complications), and for continuous indicators (oxygenation index PaO_2_/FiO_2_) we analyzed the standardized mean check (SMD). To calculate statistical heterogeneity, Cochran *Q* test and the *I*² statistic were used. Heterogeneity was considered low when the *I*² statistic was <40% and the *P*-value was >0.1. In the case of low heterogeneity, a fixed effects model was used to pool the data. If heterogeneity was high (*I*² > 40%), sensitivity analysis or subgroup analysis was used to explore potential sources of heterogeneity. We will perform subgroup analyses on different patients. Funnel plots were used to assess publication bias. Statistical software was used Review Manager (RevMan) version 5.4.1.

### 2.4. Funds

Fund sources are used for data collection, data processing, and data analysis and interpretation. All authors contributed to the article, and the authors have no prior conflicts of interest and take responsibility for the decision to submit for publication.

## 3. Results

### 3.1. Included studies and study quality

**Figure 1.**
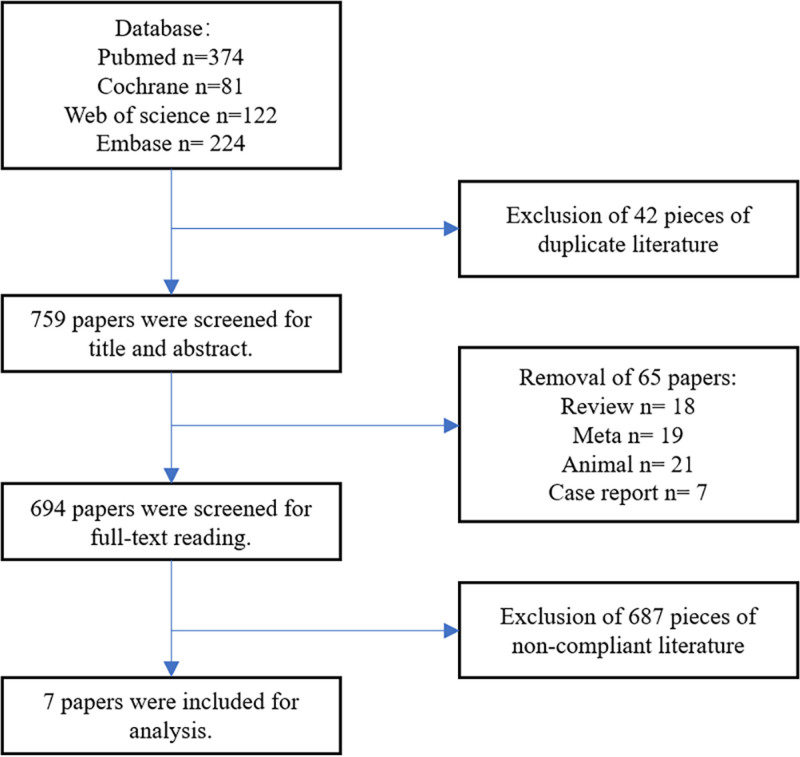
Search flow chart.

This article contains 7 studies^[18–24]^ that met the inclusion criteria and included 425 subjects (Fig. 1). Basic information on the included study articles, including year, author, and study type, was reported in Table 1. Table 2 summarized the baseline characteristics of the subjects and the procedure, among others. The included studies were assessed using the Cochrane risk of bias assessment tool and the results are available in the Appendix, Supplemental Digital Content, http://links.lww.com/MD/O230.

**Table 1:** Basic information for inclusion in the study.

Year	Author	Country	Study design
2021	Philipp^[18]^	Germany	RCT
2021	Qian^[19]^	China	RCT
2020	Felix^[20]^	Germany	RCT
2019	Liu^[21]^	China	RCT
2018	Sergio^[22]^	Brazil	RCT
2017	Nestler^[23]^	Germany	RCT
2016	He^[24]^	China	RCT

RCT = randomized controlled trial.

**Table 2:** Subjects’ baseline characteristics, trial protocol and outcomes.

Article	Surgery	Sample	Gender (M/F)	Age		BMI		EIT	Control	Outcome
				EIT	Control	EIT	Control			
Sergio 2018	20 Laparoscopic	10/10	18/22	50.7	54.2	28.3 ± 4.2	30.6 ± 4.2	ODCL	PEEP 4 cm H_2_O	1, 2
	20 Open-abdominal	10/10								2
Felix 2020	40 Laparoscopic	20/20	40/0	62.6	64.2	25.3 ± 2.3	25.6 ± 2.5	RVD	PEEP 5 cm H_2_O	1
He 2016	42 Laparoscopic	19/23	25/17	56.7	54.5	22.8 ± 3.0	24.0 ± 3.4	ROI	Best compliance	1, 2
Liu 2019	100 Thoracoscopic	50/50	44/56	70.2	69	24.3 ± 4.5	23.2 ± 3.1	ODCL	PEEP 5 cm H_2_O	1, 2
Qian 2021	80 Spinal surgery	40/40	39/41	68.2	67.7	26.81 ± 4.32	25.73 ± 3.50	CDYN	PEEP 5 cm H_2_O	1,2
Philipp 2021	69 Laparoscopic	25/44	23/46	44.9	46.5	48.2 ± 7.0	51.0 ± 9.5	RVD	PEEP 4–5 cm H_2_O	1, 2
Nestler 2017	54 Laparoscopic	27/27	20/34	44.9	46.2	43.8	53.8	RVD	PEEP 5 cm H_2_O	2

CDYN = dynamic pulmonary compliance, EIT = electrical impedance tomography, ODCL = overdistension and collapse method, ROI = best region of interest method, RVD = regional ventilation delay method.

Outcome 1, oxygenation index (PaO_2_/FiO_2_); Outcome 2, postoperative pulmonary complications.

### 3.2. Oxygenation index PaO_2_/FiO_2_

**Figure 2.**
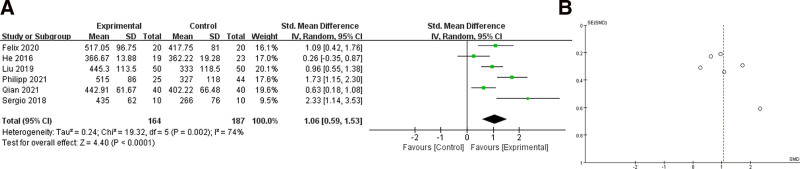
PaO_2_/FiO_2_ ratio analysis: (A) shows a forest plot with SMD = 1.06 (95% CI 0.59–1.53); (B) shows a funnel plot. SMD = standardized mean check.

**Figure 3.**
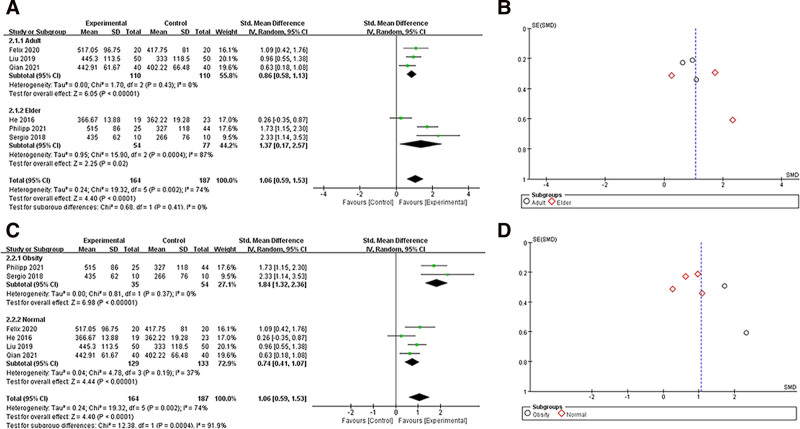
PaO_2_/FiO_2_ ratio subgroup analysis: (A) shows a forest plot for age subgroup analysis; (B) shows a funnel plot for age subgroup analysis; (C) shows a forest plot for BMI subgroup analysis; (D) shows a funnel plot for BMI subgroup analysis.

A total of 6 studies reported oxygenation index (PaO_2_/FiO_2_) results,^[18–22,24]^ and data were collected and included in the analysis. Due to the large heterogeneity of the data after the combined analysis (*I*^2^ > 40%), we chose a random-effects model for the analysis. For the combined analysis, SMD = 1.06 (95% CI 0.59–1.53), see Figure 2. The difference was statistically significant (*P* < .0001), indicating that patients had a higher oxygenation index when individual PEEP titration was performed with EIT. To determinate the influence of age and weight, we divided the patients into an elder group (age > 60) and an adult group (age 18–60); then obese group (BMI > 28) and a normal group (BMI < 28) for subgroup analysis. The results were as follows (Fig. 3), with SMD = 0.86 (95% CI 0.58–1.13) in the adult group and SMD = 1.37 (95% CI 0.17–2.57) in the elder group, with decreased heterogeneity in the adult group (*I*^2^ = 0%) but increased heterogeneity in the elder group (*I*^2^ = 87%). In the meanwhile, SMD = 1.84 (95% CI 1.32–2.36) in the obese group and SMD = 0.74 (95% CI 0.41–1.07) in the normal group, heterogeneity decreased in both groups. Moreover, the results of subgroup analysis were statistically significant both for age and weights (*P* < .05).

### 3.3. Postoperative pulmonary complications

**Figure 4.**
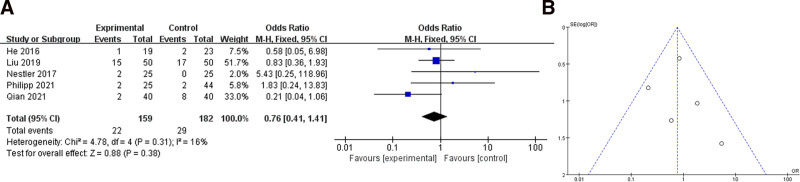
Analysis of postoperative pulmonary complications: (A) shows a forest plot; (B) shows a funnel plot.

**Figure 5.**
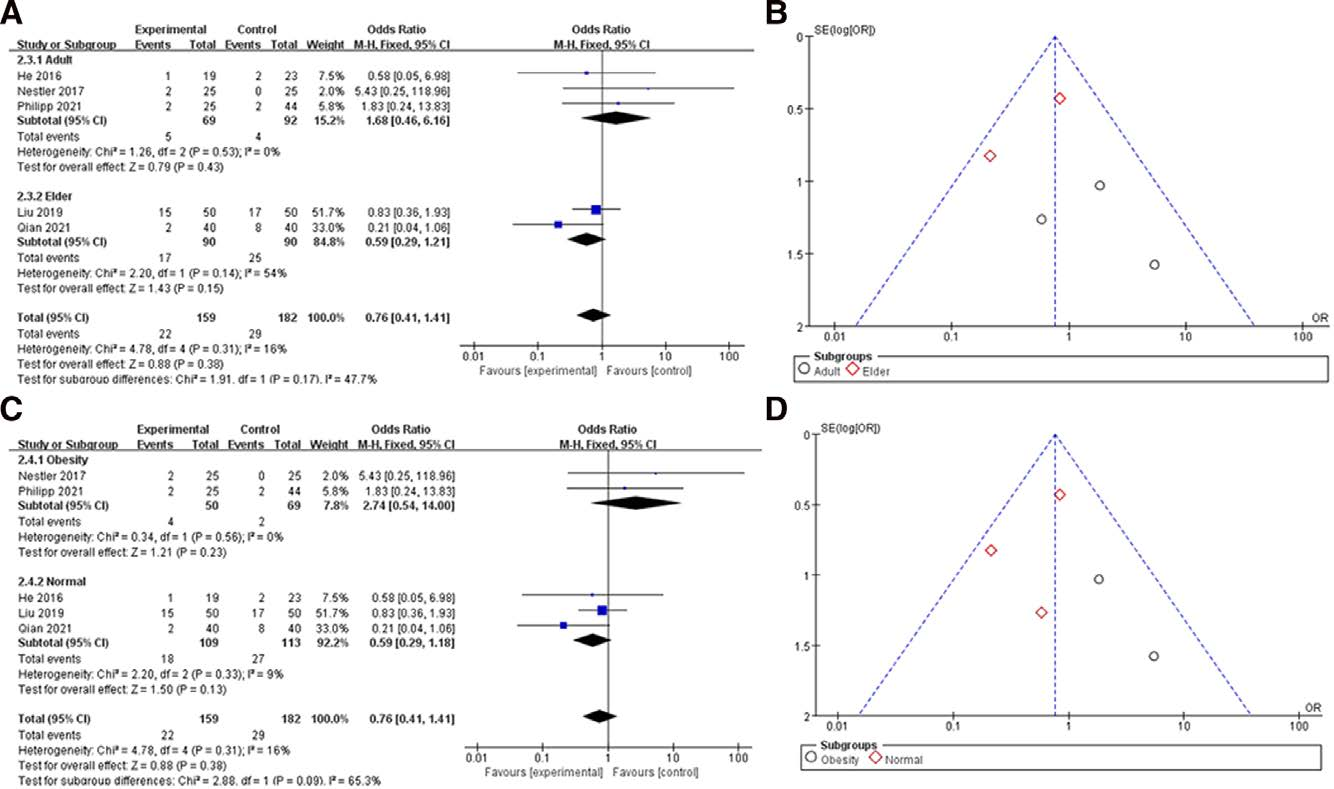
Subgroup analysis of postoperative pulmonary complications: (A) shows a forest plot of age subgroup analysis; (B) shows a funnel plot of age subgroup analysis; (C) shows a forest plot of BMI subgroup analysis; (D) shows a funnel plot of BMI subgroup analysis.

Six of the seven studies reported the outcome of postoperative pulmonary complications.^[18,19,21–24]^ Forty samples in Sergio study had not reported the postoperative pulmonary complications and therefore were not shown in the figure. We analyzed the included articles showing a total SMD = 0.76 (95% CI 0.41–1.41) and *I*^2^ = 16%, so a fixed effects model was used for the analysis. The results shown were not statistically significant (*P* > .05), see Figure 4. After that we also divided them into the elder group (age > 60) and an adult group (age 18–60), obese group (BMI > 28) and a normal group (BMI < 28) for subgroup analysis, see Figure 5. Adult group SMD = 1.68 (95% CI 0.46–6.16) and elder group SMD = 0.56 (95% CI 0.29–1.21), *I*^2^ decreased in adult group but increased in elder group. For BMI subgroups, SMD = 2.74 (95% CI 0.54–14.00) in the obese group and SMD = 0.59 (95% CI 0.29–1.88) in the normal group, both with decreased *I*^2^. The results shown were still not statistically different between the 2 subgroups (*P* > .05).

## 4. Discussion

Our results showed that after personalized intraoperative titration of patients using electrical impedance tomography, PaO_2_/FiO_2_ values were significantly improved compared to other modalities of PEEP titration, but for the occurrence of postoperative pulmonary complications, there was no significant difference between the EIT and control groups, and both groups had similar efficacy. Two recent comparisons of EIT-titrated PEEP modalities versus other PEEP modalities have demonstrated the advantages of EIT titration,^[25,26]^ but the present study included more articles as well as more surgical modalities in an attempt to increase the broad applicability of this study.

Currently, individualized PEEP titration is guided by driving pressure (DP), transpulmonary pressure (PL), lung compliance, pressure–volume curves (P–V curves), and visualization techniques. DP-guided individualized PEEP titration refers to the adjustment of PEEP during ventilation, and the calculation of PEEP at each PEEP level based on (DP = plateau pressure ‐ PEEP). DP, and the PEEP corresponding to the lowest DP is the optimal PEEP. Decremental titration is more effective in reducing DP and improving oxygenation.^[27]^ The minimum PEEP at end-expiratory PL > 0 cm H_2_O (1 cm H_2_O = 0.098 kPa) and end-inspiratory PL < 25 cm H_2_O is often used as the optimal PEEP for PL-guided individualized PEEP titration. However, due to the high requirements of esophageal manometry, complex operation and many influencing factors, it is less used in the perioperative period. The P–V curve takes the airway pressure as the horizontal axis and the end-expiratory lung volume as the vertical axis, which is in the shape of “S,” and the low inflection point of the curve is the starting point of the reexpansion of the atrophied alveoli. Individualized PEEP titration is to adjust the PEEP when the tidal volume is fixed, calculate the static or dynamic pulmonary compliance, and the PEEP that corresponds to the maximum of the 2 is the optimal PEEP. The dynamic pulmonary compliance is often the main focus in clinical practice, but due to individual differences and uneven ventilation delay in the lung region, the optimal PEEP needs to be selected according to the actual situation in the operation. P–V curve guidance Individualized PEEP titration is based on a pressure slightly greater than the low inflection point as the optimal PEEP, which allows more alveoli to maintain an open state and avoids atrophy injuries caused by cyclic opening and closing of alveoli.

The titration of EIT in the included studies included the overdistension and collapse method, the regional ventilation delay method, the optimal ROI method, and the dynamic lung compliance (CDYN) method. In addition to these there were end-expiratory lung impedance method^[9,28]^ and GI method.^[28]^ Based on the differences in the “best PEEP” titrated by different EIT methods,^[28]^ there was a lack of uniform criteria for the selection of methods. Several clinical studies had demonstrated the safety and feasibility of titration of PEEP in ARDS patients based on the OD/CL method of local compliance change.^[29–33]^ The combination of 2 or more EIT parameters may help to provide a more comprehensive assessment of ventilation and better guide the setting of “optimal PEEP.” Clinical studies had supported the OD/CL method and GI as common reference indicators for PEEP titration in animal models of ARDS and clinical cases, respectively.^[33,34]^ Nestler et al^[22]^ showed that the use of the EIT technique to calculate the local ventilation delay index and set the lowest PEEP matching the local ventilation delay index as the optimal PEEP compared with a fixed PEEP (5 cm H_2_O) group improved the performance of general anesthesia.

PPCs were another major outcome indicator in our study, and by analysis we found that after PEEP titration with EIT, it did not show an advantage in PPCs compared to the conventional PEEP setting. In fact, in a previous multicenter study (PROVHILO trial) there was no difference in postoperative complications and prognosis between the use of high and low PEEP values in laparotomy.^[35]^ Lung protective ventilation strategies are increasingly valued by anesthesiologists. Low tidal volumes and pulmonary resuscitation have been initially demonstrated with lung protective ventilation strategies, but how to set optimal PEEP remains controversial. Due to individual patient differences, fixed PEEP cannot be satisfied in all patients. Low PEEP values may lead to pulmonary atelectasis and ground oxygenation index, while high PEEP values can lead to alveolar injury and severely compromise the patient’s circulatory function. Optimal PEEP can be defined as a PEEP value that improves perioperative lung function and increases oxygenation with maximum oxygenation, minimum driving pressure, maximum respiratory compliance, minimum alveolar dead space volume, and less hemodynamic fluctuations.^[36]^ But optimal PEEP did not reduce PPCs incidence

Various factors influence PaO_2_/FiO_2_ and PPCs, including ventilator setup parameters, preoperative patient lung function and surgical approach, among others. In successive studies, increasing age has been correlated with PPCs regardless of which PPCs’ accuracy by criteria is used, and age is a predictor of PPCs.^[37]^ Pulmonary insufficiency itself can affect PaO_2_/FiO_2_ values, and periodic pulmonary resuscitation and insufficiency of resuscitation may lead to PPCs, especially in obese patients.^[38–40]^ Therefore, in our study, age and BMI data were extracted and analyzed for heterogeneity. We also performed a subgroup analysis (for age and weight) and found that the oxygenation index remained significantly higher after PEEP titration with EIT than in the conventional PEEP group. Postoperative pulmonary complications subgroup analysis remained no difference between the 2 groups. This suggests that our findings were not influenced by patient baseline values or study modality and were true for individual PEEP titration performed by EIT. Interestingly, there was a significant decrease in heterogeneity between the 2 groups when performing the BMI subgroup analysis, suggesting a high degree of consistency in the way interventions and data collection were performed in these studies for patients with the same similar BMI. EIT-guided PEEP is equally beneficial in elderly and obese patients, both improving oxygenation indices, but the relationship with PPCs is not clear. After intraoperative titration of PEEP with EIT for mechanical ventilation, it does not significantly improve the incidence of postoperative complications in patients, but it can increase the oxygenation index of the patient during the operation, and this index is equally applicable in the surgery of the elderly, and in the surgery of obese patients.

The results of our study have some limitations. First, the small sample size of 7 studies included in our study may bias the results of the analysis. In addition, most of the articles included in our study did not specify the blinding method, and these flaws may affect the authenticity of the findings. Finally, the study outcomes in this article were limited, including only the oxygenation index and postoperative pulmonary complications. Expanding the scope of the findings could provide a more comprehensive understanding of the efficacy and safety of the intervention, which will be the direction of our subsequent research.

In conclusion, our findings suggested that EIT-guided individual PEEP titration improved the perioperative oxygenation index (PaO_2_/FiO_2_) in surgical patients compared with other PEEP titration strategies, but there was no significant difference in the occurrence of PPCs. The results of this study may provide a reference for clinical practitioners to help fully evaluate the selection of an appropriate ventilation strategy for perioperative patients.

## Author contributions

**Conceptualization:** Lifang Chen, Kang Yu.

**Formal analysis:** Kang Yu, Jiaojiao Yang, Xue Han, Lei Liu.

**Funding acquisition:** Huihui Miao.

**Investigation:** Tianzuo Li.

**Writing – original draft:** Lifang Chen, Kang Yu.

**Writing—review & editing:** Tianzuo Li, Huihui Miao.

## Supplementary Material


