# Supplementary figures and images for: Electrical impedance tomography-guided positive end-expiratory pressure titration for perioperative oxygenation and postoperative pulmonary complications: A systematic review and meta-analysis
Source: Medicine (Baltimore). 2024 Dec 27;103(52):e40357. doi: 10.1097/MD.0000000000040357 (PMC11688048; doi:10.1097/MD.0000000000040357)

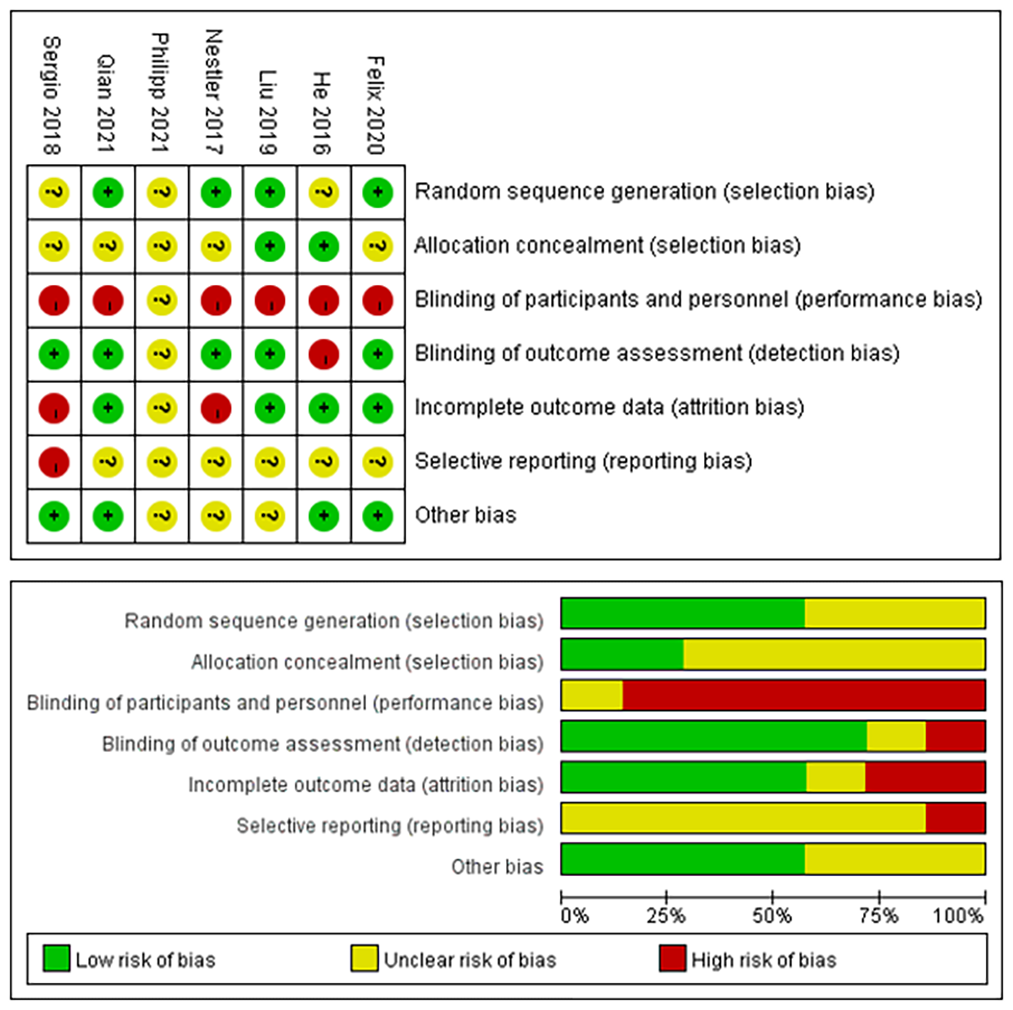

Supplement: Supplementary file 1 [file medi-103-e40357-s001.tif]
